# Supplementary material for: FAF1 phosphorylation by AKT accumulates TGF-β type II receptor and drives breast cancer metastasis
Source: Nat Commun. 2017 Apr 26;8:15021. doi: 10.1038/ncomms15021 (PMC5414047; doi:10.1038/ncomms15021)
Supplement: Supplementary Information — Supplementary Figures, Supplementary Tables and Supplementary Reference [file ncomms15021-s1.pdf]

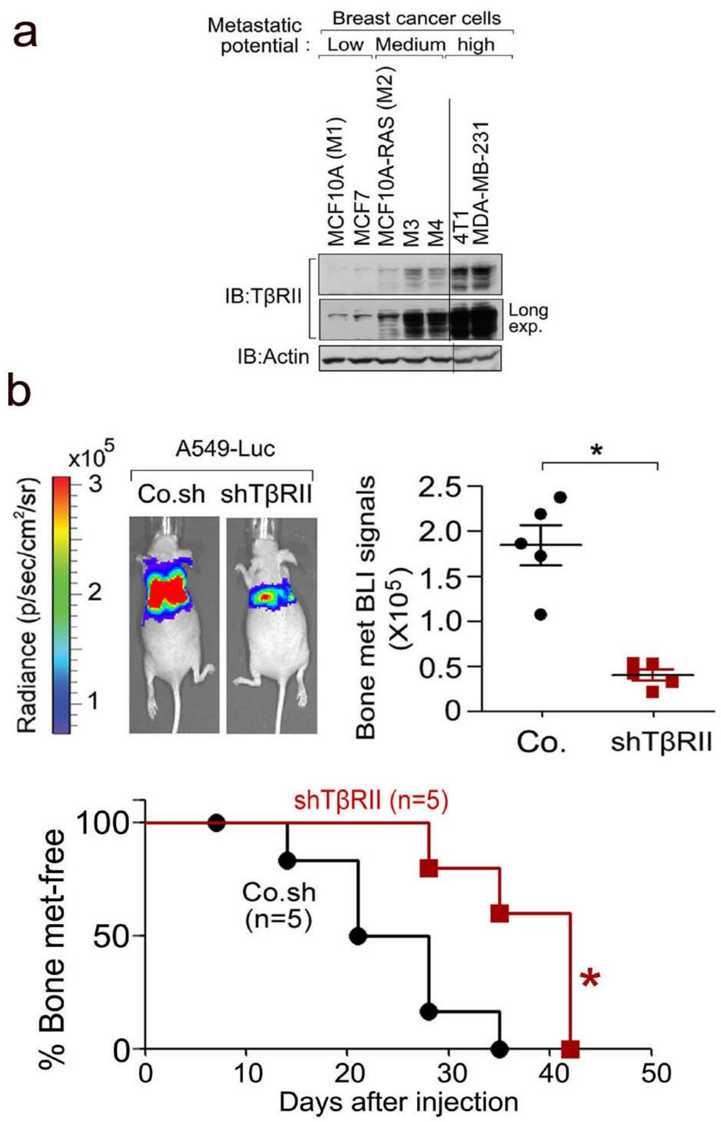

Supplementary Figure 1. TβRII expression increases and is required for aggressive cancer metastasis.

(a) Immunoblot (IB) analysis of TβRII protein levels in breast cancer cell lines with different potential (low, medium and high) of metastasis in xenograft models.

(b) Bioluminescent imaging (BLI) of representative mice from each group injected with control (Co.) or MDA-MB-231 cells stably depleted of TβRII (shTβRII). Images were captured at week 6 following left heart ventricle injection. Dorsal images are shown. The BLI signal of every mouse in each experimental group is shown in the right panel. The percentage of bone metastasis free mice in each group in time is shown in the bottom panel.

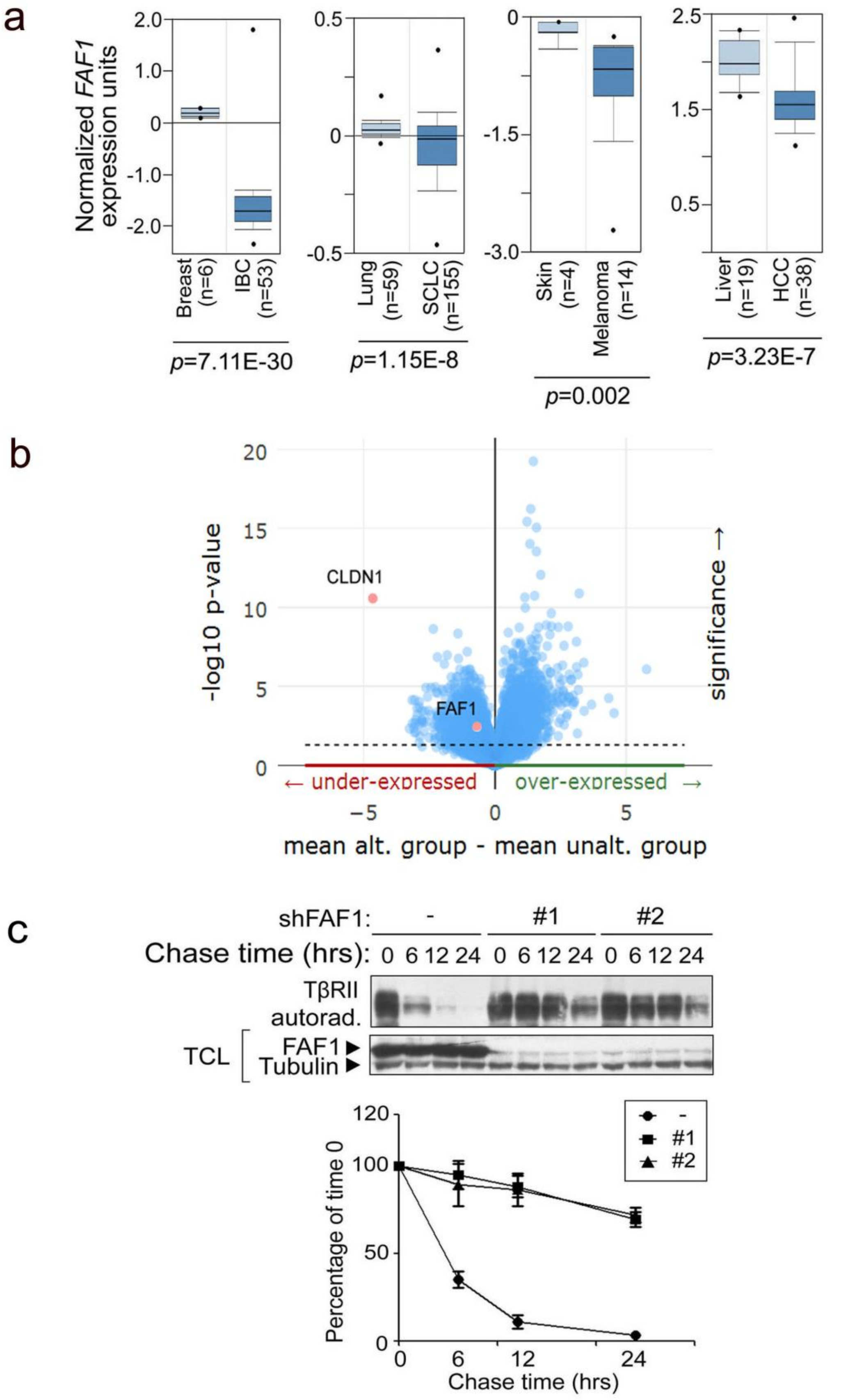

a
Normalized FAF1 expression units
2.0
1.0
0
-1.0
-2.0
Breast (n=6)
IBC (n=53)
p=7.11E-30
0.5
0
-0.5
Lung (n=59)
SCLC (n=155)
p=1.15E-8
0
-1.5
-3.0
Skin (n=4)
Melanoma (n=14)
p=0.002
2.5
1.5
0.5
Liver (n=19)
HCC (n=38)
p=3.23E-7
b
-log10 p-value
significance →
CLDN1
FAF1
← under-expressed
over-expressed →
mean alt. group - mean unalt. group
c
shFAF1:
-
#1
#2
Chase time (hrs):
0 6 12 24
TβRII autorad.
TCL
FAF1 ▶
Tubulin ▶
Percentage of time 0
120
100
50
0
0
6
12
24
Chase time (hrs)
- #1 #2

Supplementary Figure 2. FAF1 expression is downregulated in multiple human cancers, and FAF1 controls TβRII turnover.

(**a**) Oncomine [1] box plots of FAF1 expression levels in multiple human advanced cancers vs. normal tissues; mean±SD, $p<0.01$.

(**b**) Volcano plot showed the Claudin 1 (CLDN1) low patients have significant under-expressed FAF1 expression (Z score of CLDN1<2.5). Data from the TCGA breast cancer database.

(**c**) [^{35}S]-methionine labeling and pulse-chase analysis of TβRII in control and MDA-MB-231 cells with stable knockdown of FAF1 by two independent shRNA (#1 and #2). The amount of precipitated labeled protein after the chase was expressed as a percentage of the amount present at the beginning of the chase (time 0) and is shown in the lower panels. The results are shown as the mean±SD of three independent sets of experiments. Tubulin immunoblot in total cell lysate (TCL) is included as a loading control.

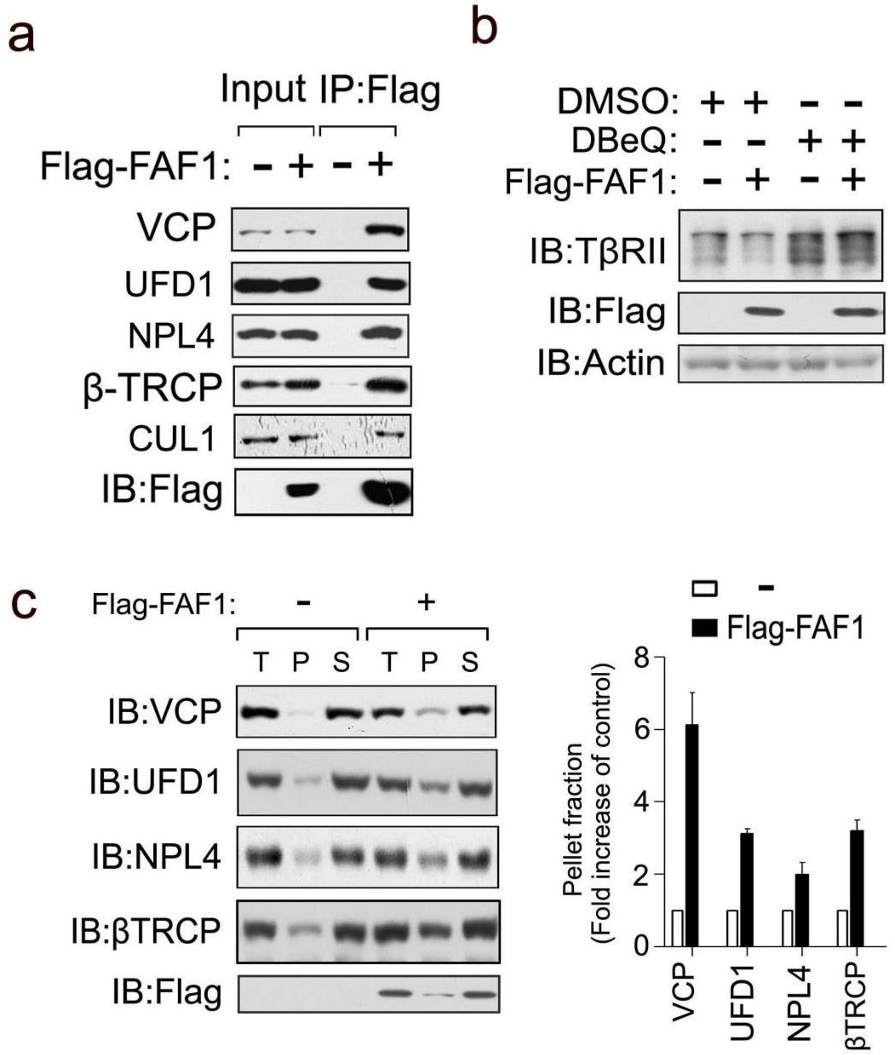

Supplementary Figure 3. FAF1 recruits the VCP-E3 ligase complex to target TβRII turnover.

(**a**) HEK293T cells were transfected with or without Flag-FAF1. The cells were then harvested for immunoprecipitation (IP) and immunoblot (IB) analysis.

(**b**) Control and MDA-MB-231 cells with or without stable Flag-FAF1 expression were treated with *N*2,*N*4-*Bis* (phenylmethyl)-2,4-quinazolinediamine (DBeQ) (10 μM) or solvent DMSO control overnight as indicated. Cells were then harvested for IB analysis.

(**c**) IB of the total (T), pellet (P) and soluble (S) fractions of control and Flag-FAF1-expressing HEK293T cells. The band intensity of the pellet fraction was quantified and is shown in the right panel. The band intensity in the control cells was set at 1. The results are shown as the mean±SD of three independent sets of experiments.

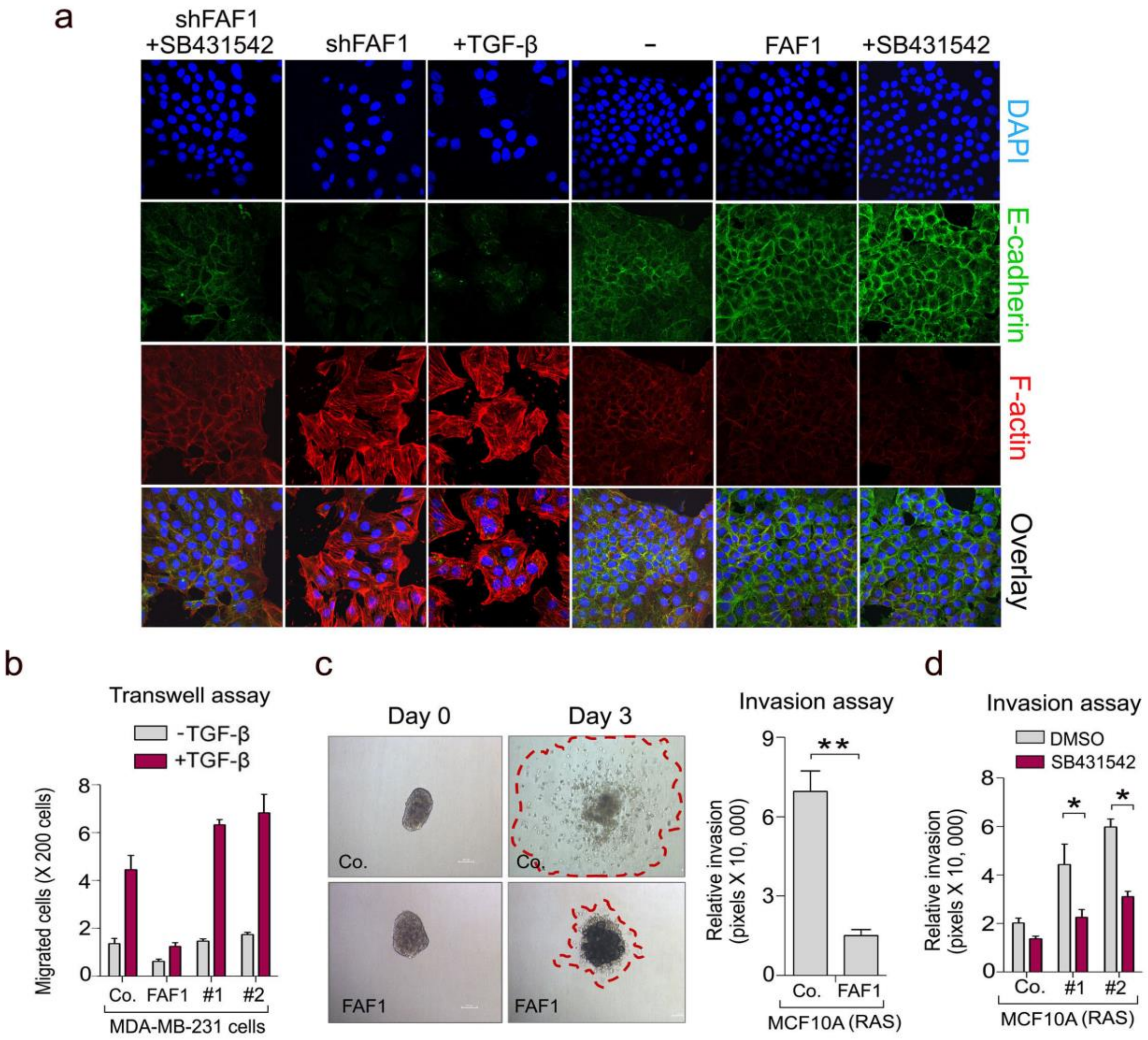

Supplementary Figure 4. FAF1 inhibits EMT, migration and invasion *in vitro*.

(**a**) Immunofluorescence with antibody specific for E-Cadherin, phalloidin for filamentous (F)-actin and 4, 6-diamidino-2-phenylindole (DAPI) staining of control and HaCaT cells stably depleted of or overexpressing FAF1 and treated with SB431542 (10 μM) or TGF-β (1 ng ml^{-1}) for 72 h. Scale bar, 10 μm.

(**b**) Transwell assay performed with MDA-MB-231 cells stably overexpressing FAF1 or depleted of FAF1 by shRNA (#1 and #2), and with or without TGF-β treatment (5 ng/ml) for 8 h. Co., control non-coding shRNA.

(**c**) 3D spheroid invasion assay of control and stable FAF1-expressing MCF10A-RAS cells. Borders are indicated by a dashed red line. Representative images at day 0 and day

3 of each group are shown (Left panel). Relative invasion is quantified. The data are presented as the mean±SD of three independent sets of experiments (Right panel). ** $p<0.01$.

(**d**) 3D spheroid invasion assay of control and FAF1-depleted MCF10A-RAS cells (#1 and #2) with or without SB431542 (10 μM) treatment. Relative invasion is quantified and shown. The data are presented as the mean±SD of three independent sets of experiments.

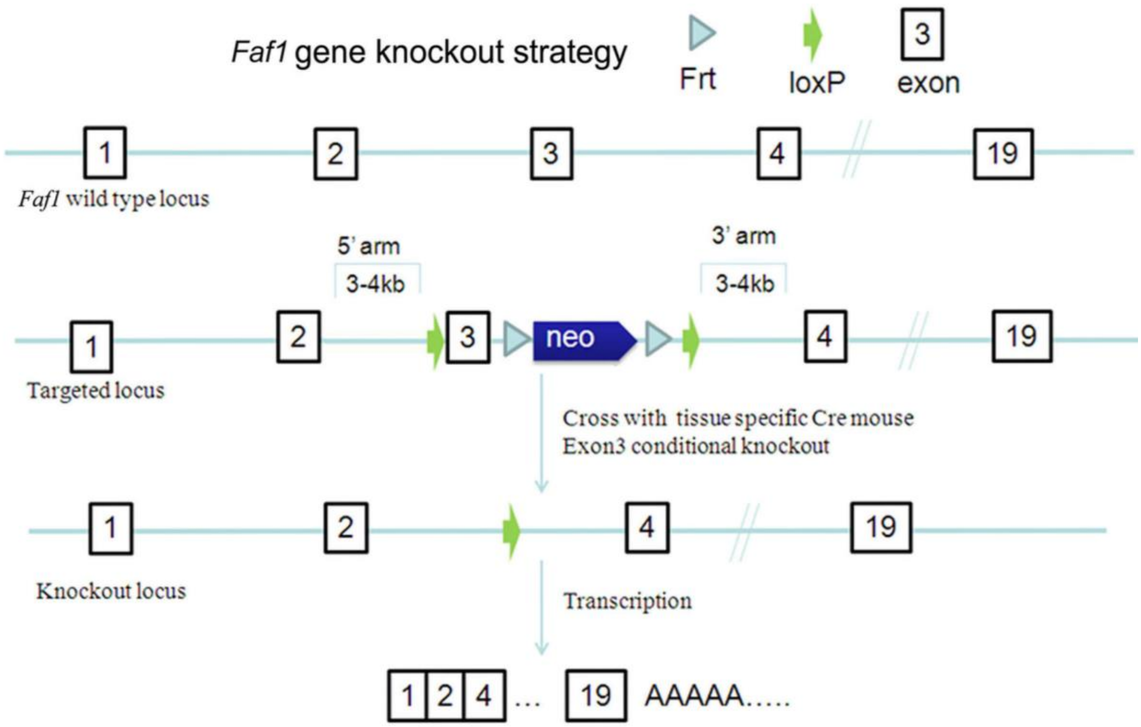

Supplementary Figure 5. Schematic diagram of the Faf1 gene-knockout strategy (also described in the "Materials and Methods" section).

Faf1$^{flox/+}$ mice (C57BL/6J) were generated by standard homologous recombination at Shanghai Biomodel Organism, Shanghai, China. In these mice, *Faf1* exon 3, which encodes the signal peptide, was flanked by *loxP* sequences. *Faf1$^{flox/+}$* mice were then mated to *EIIa-Cre* transgenic mice (FVB/N; Jackson Laboratory), in which the adenovirus *EIIa* promoter directs expression of the Cre enzyme in early mouse embryos (two- to eight-cell stage) to achieve homologous recombination between *LoxP* sites and to trigger the deletion of exon 3 in all cells of the developing animal, including the germ cells that transmit the genetic alteration to progeny. Deletion of exon 3 results in frame shift and disrupts its open reading frame (ORF), leading to the loss of *Faf1* expression. The first generation of *EIIa-Cre*; *Faf1$^{flox/+}$* mice could be chimeric due to the mosaic activity of Cre recombinase. Therefore, the chimeric offspring were backcrossed with C57BL/6J mice to generate *Faf1$^{+/-}$* mice, which then were intercrossed for production of *Faf1*-deficient (*Faf1$^{-/-}$*) mice.

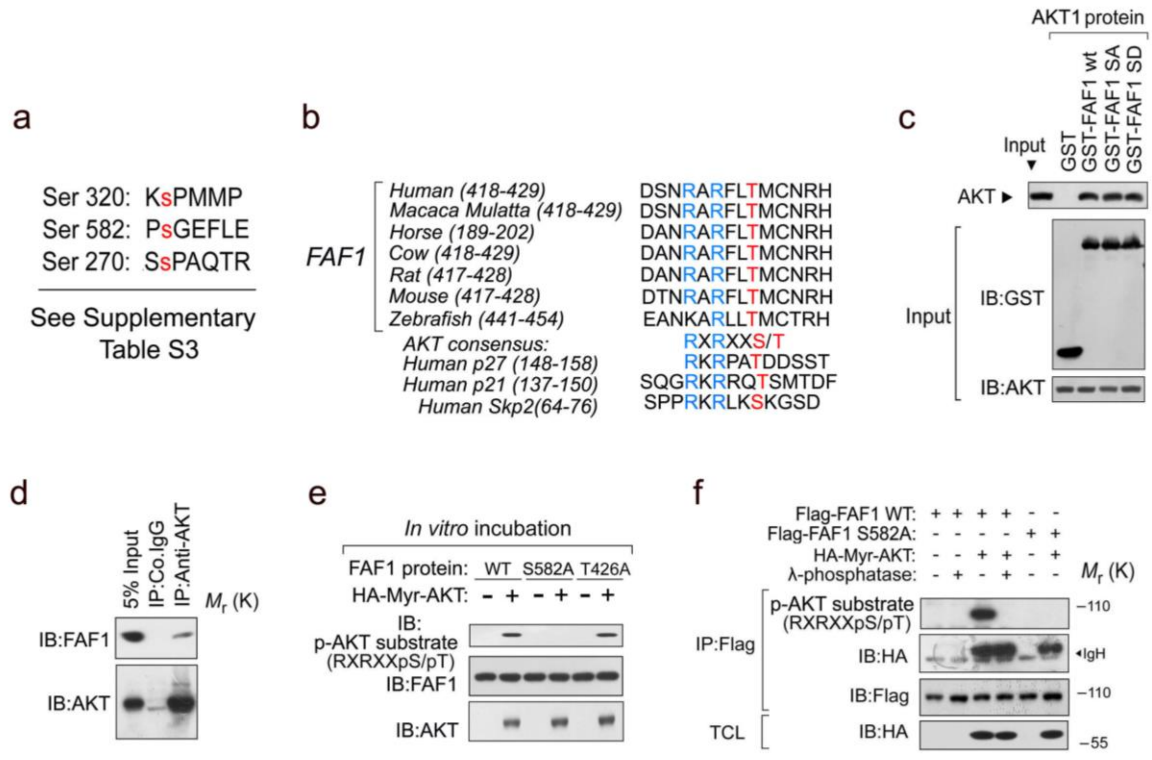

Supplementary Figure 6. AKT phosphorylates FAF1 on Ser 582.

(**a**) The peptide sequences of FAF1 phosphorylation sites and neighboring motifs identified in a mass spectrometry study.

(**b**) Sequence alignment of the other conserved AKT phosphorylation site within FAF1 orthologs (human FAF1 Thr 426) from different species and the known AKT phosphorylating proteins CDK inhibitors p27, p21, and S-phase kinase-associated protein 2 (Skp2).

(**c**) *In vitro* purified FAF1 wt and mutants (S582A and S582D) associated with AKT as measured by pulldown experiment (top panel). Input controls are shown by immunoblot (IB) for GST and AKT.

(**d**) Immunoblot (IB) analysis of input and immunoprecipitated material derived from HEK293T cells. 5% of the total cell lysate was loaded as input.

(**e**) AKT phosphorylates FAF1 *in vitro* at Ser 582. HA–Myr-AKT was transfected into 293T cells, recovered by anti-HA immunoprecipitation and incubated with 5 µg of the indicated GST–FAF1 proteins in the presence of unlabeled ATP. The kinase reaction products were resolved by SDS–PAGE, and phosphorylation was detected by the phospho-AKT substrate antibody that recognizes the RXRXXpS(pT) motif.

(f) IB of total cell lysate (TCL) and immunoprecipitants derived from HEK293T cells transfected with Flag-FAF1 WT, S582A mutant or HA-Myr-Akt plasmids as indicated and treated with or without λ-phosphatase for 1 h after lysing.

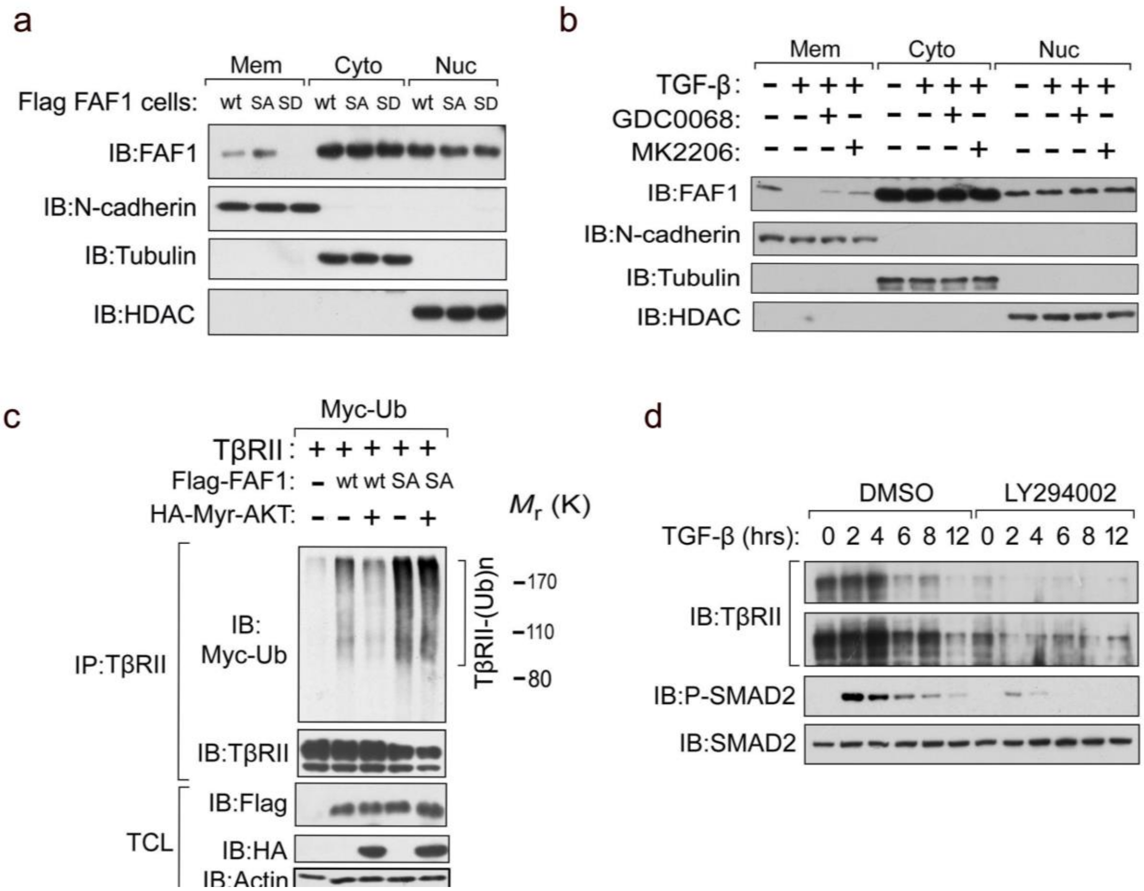

Supplementary Figure 7. Phosphorylation of FAF1 Ser 582 inhibits its membrane localization and antagonizes FAF1's effect on TβRII turnover.

(**a**) Immunoblot (IB) of the membrane (Mem), cytoplasm (Cyto) and nuclear (Nuc) fractions derived from HeLa cells transfected with Flag-FAF1 wt, S582A (SA) or S582D (SD) expression plasmids. Controls to confirm the purity of the different fractions were taken along.

(**b**) HeLa cells treated with or without selective AKT inhibitors GDC0068 (5 μM) or MK2206 (5 μM) as indicated for 1 h prior to TGF-β (5 ng/ml) stimulation. Cells were harvested for membrane (Mem), cytoplasm (Cyto) and nuclear (Nuc) fragment extraction and IB analysis.

(**c**) IB of TCL and immunoprecipitants derived from Myc-Ub stably expressing HEK293T cells transfected with TβRII along with Flag-FAF1 wt or SA mutant and Myr-HA-Akt expression plasmids, as indicated.

(**d**) IB of TCL of bone metastatic MDA-MB-231 cells treated with control DMSO or selective Phosphatidylinositol-3-Kinase (PI3K) inhibitor LY294002 (50 μM) for 12 h and TGF-β (5 ng ml^{-1}) at the indicated time points.

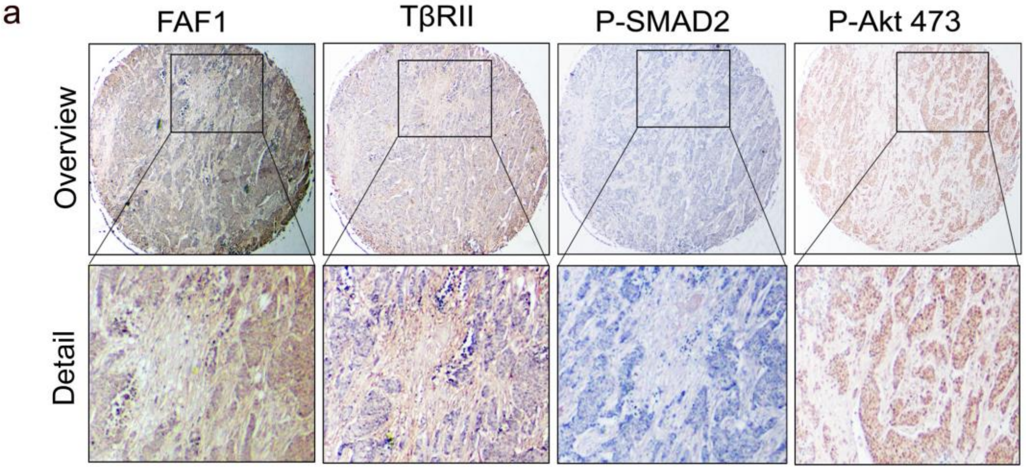

b

	FAF1-low	FAF1-high	Total
TβRII-low	17	33	50
TβRII-high	55	6	61
Total	72	39	111

X^2= 38.03 P=3.5E-08

	FAF1-low	FAF1-high	Total
P-S2-low	47	35	82
P-S2-high	25	4	29
Total	72	39	111

X^2= 7.85 P= 0.001

	FAF1-low	FAF1-high	Total
P-AKT low	29	21	50
P-AKT high	43	18	61
Total	72	39	111

X^2= 1.882 P= 0.05

c

	TβRII-low	TβRII-high	Total
P-S2-low	49	33	52
P-S2-high	1	28	59
Total	50	61	111

X^2= 27.44 P=3.37E-06

	TβRII-low	TβRII-high	Total
P-AKT low	29	23	52
P-AKT high	18	41	59
Total	47	64	111

X^2= 7.22 P= 0.01

d

	P-S2-low	P-S2-high	Total
P-AKT low	42	8	50
P-AKT high	40	21	61
Total	80	29	111

X^2= 4.83 P= 0.0034

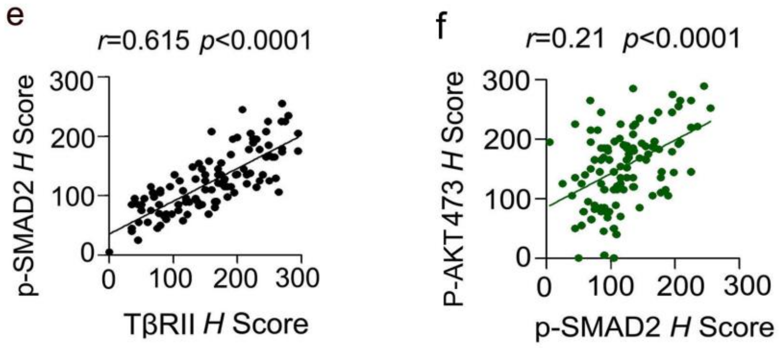

Supplementary Figure 8. Loss of FAF1 correlates with increased TβRII as well as downstream p-SMAD2 and p-AKT expression in patients, and the SMAD and AKT signals are co-activated in breast cancer patients.

(**a**) Representative images of each antibody staining are shown. Up panel: objective, 5×; Low panel: objective, 25×.

(**b**) Case number of specimens showing low or high FAF1 expression in relation to the expression levels of TβRII, p-SMAD2 (P-S2) and p-AKT Ser 473 (P-AKT).

(**c**) Case number of specimens showing low or high TβRII expression in relation to the expression level of P-SMAD2 (P-S2) and P-AKT Ser 473 (P-AKT).

(**d**) Case number of specimens showing low or high P-SMAD2 (P-S2) expression in relation to the expression of P-Akt Ser 473 (P-AKT).

(**e**) The H score of each tissue sample for TβRII and P -SMAD2 was calculated, and the two-tailed significance is shown.

(**f**) The H score of each tissue sample for P-SMAD2 and P-AKT 473 was calculated, and the two-tailed significance is shown.

Figure 1b

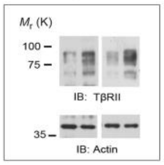

Figure 1c

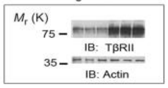

Figure 1e

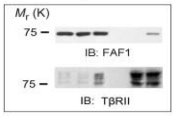

Figure 1g

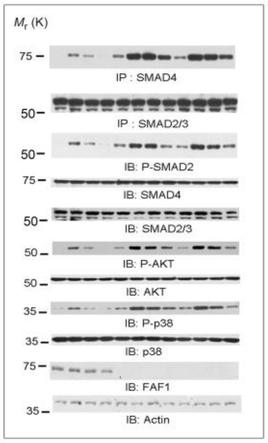

Figure 1h

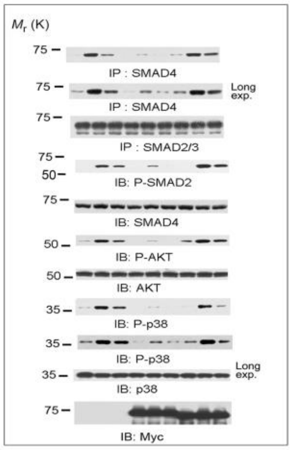

Figure 2a

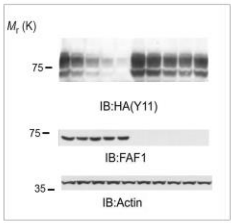

Figure 2c

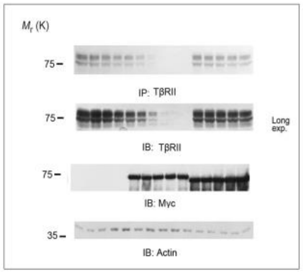

Figure 2d

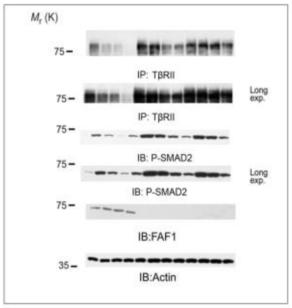

Figure 3e

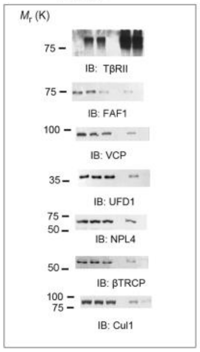

Figure 3f

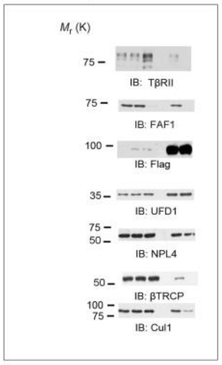

Figure 3g

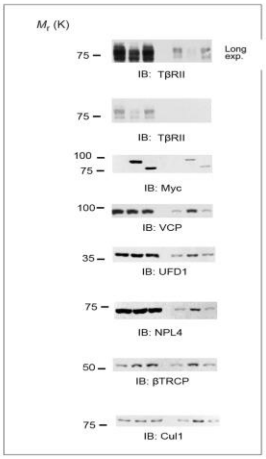

Figure 3h

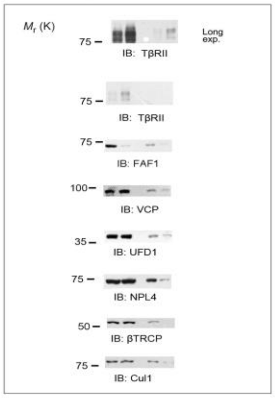

Figure 3i

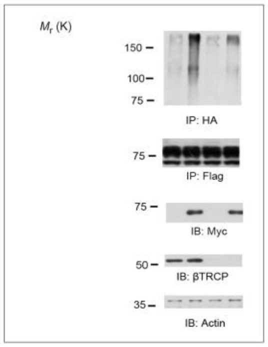

Figure 3j

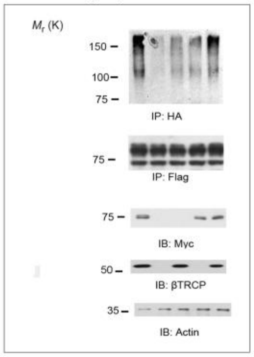

Figure 3k

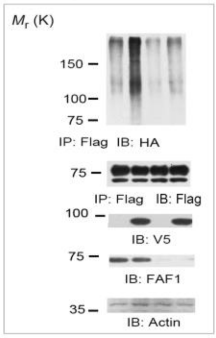

Figure 4b

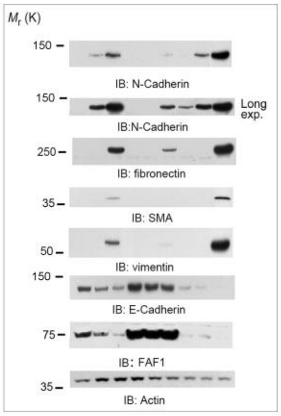

Figure 5b

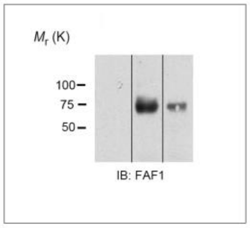

Figure 5c

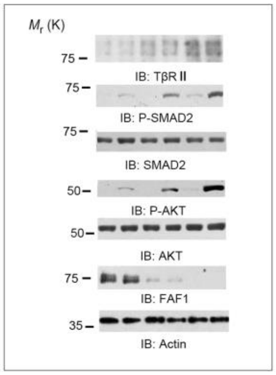

Figure 6c

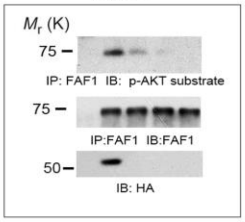

Figure 6d

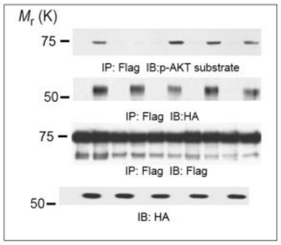

Figure 6e

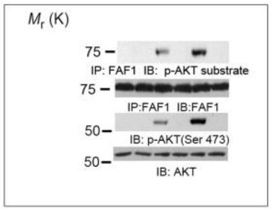

Figure 6f

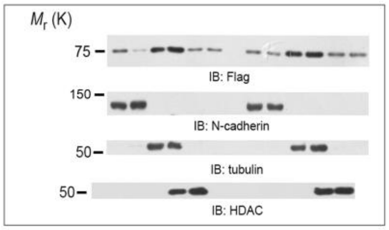

Figure 6g

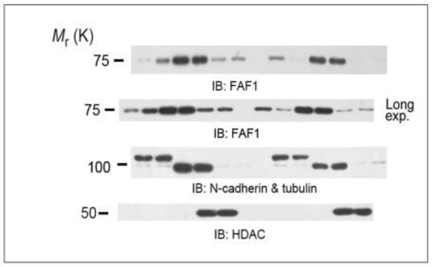

Figure 6h

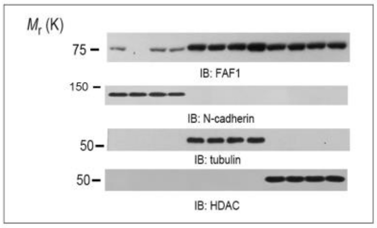

Figure 7a

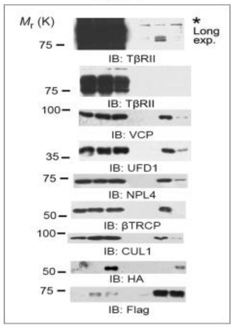

Figure 7b

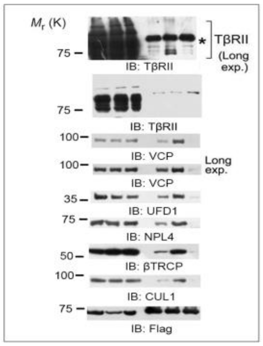

Figure 7c

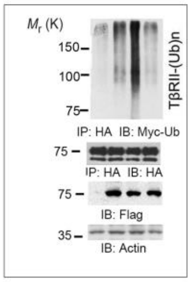

Figure 7d

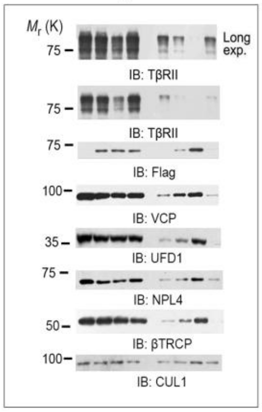

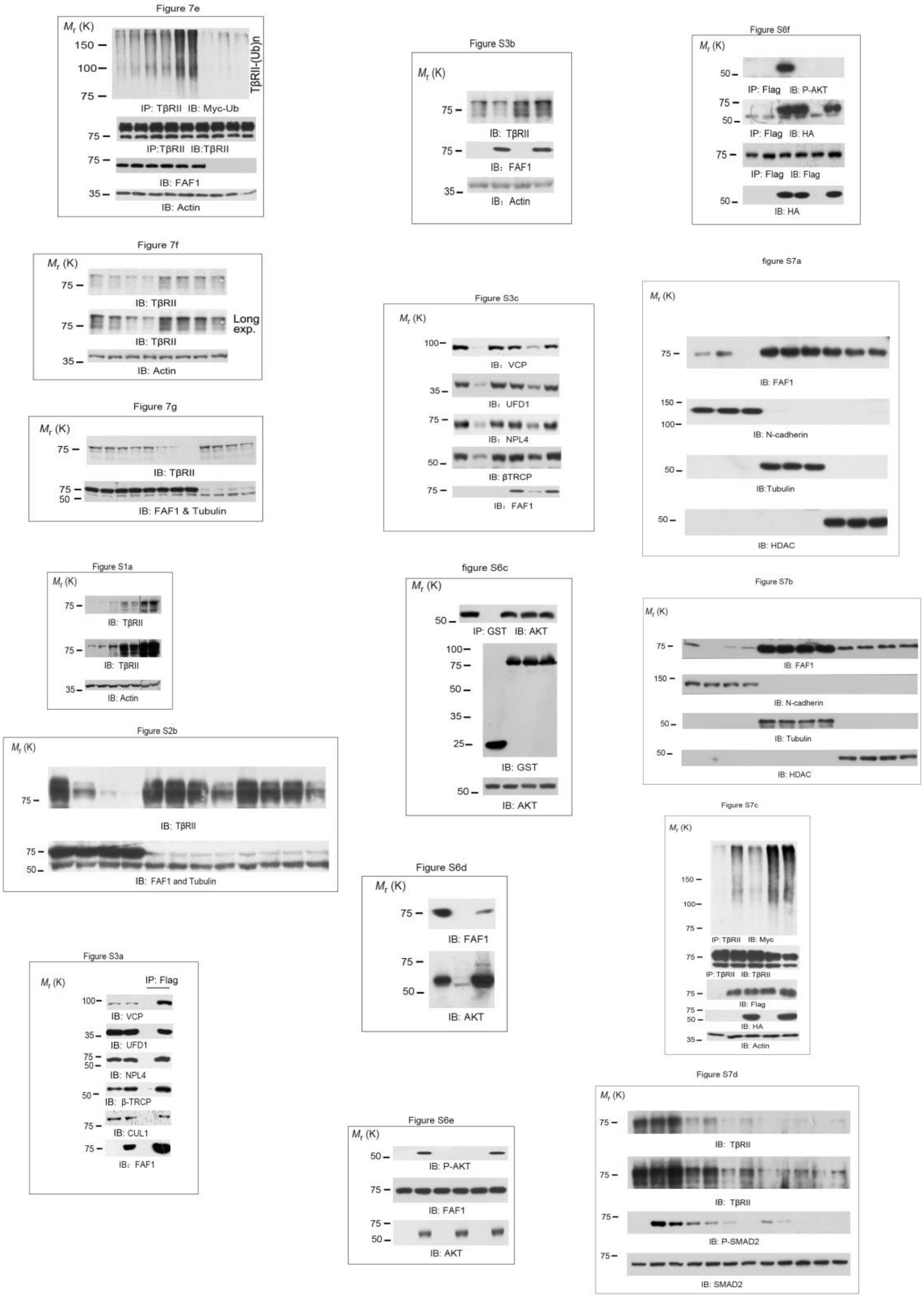

Supplementary Figure 9. Uncropped scans of western blots included in main figures.

Supplementary Table 1, Potential specific binding protein of TβRII. (These proteins were only identified in sample B with at least two peptide assignments. See Supplementary Data 1 .)

proteins	#Unique peptides	Sequence coverage
FAF1_HUMAN	12	20
E7ET15_HUMAN	4	6.5
C9J9K3_HUMAN	4	23.1
ECM29_HUMAN	4	3.3
PSD11_HUMAN	3	8.5
ACSL4_HUMAN	3	7.2
ERAL1_HUMAN	3	10.5
I3L3C4_HUMAN	3	22.2
HXK1_HUMAN	3	3.5
E9PMW7_HUMAN	3	38.5
B7Z1R5_HUMAN	3	7.5
BAG6_HUMAN	3	4.7
B4DR63_HUMAN	3	14.7
F5H335_HUMAN	3	2.6
M0R001_HUMAN	3	33.3
CDC5L_HUMAN	3	5.6
B7Z7A3_HUMAN	3	7.2
PSDE_HUMAN	2	12.6
E9PJR8_HUMAN	2	11.2
FADS2_HUMAN	2	6.7
B7Z7A9_HUMAN	2	8.2
H3BR04_HUMAN	2	19.8
H3BQ34_HUMAN	2	14.6
PSA3_HUMAN	2	8.9
H0YKT8_HUMAN	2	9.9
CTR1_HUMAN	2	4.8
B4DZI8_HUMAN	2	3.2
F8W9B4_HUMAN	2	12.9
STAT1_HUMAN	2	3.1
PRS6B_HUMAN	2	13.4
B4DFL2_HUMAN	2	6.8
Q7Z6V3_HUMAN	2	15.4
D6RED7_HUMAN	2	16.8
E9PKZ0_HUMAN	2	13.2

F5H4B1_HUMAN	2	5.5
ASPH_HUMAN	2	5.3
KRR1_HUMAN	2	7.1
SPTN1_HUMAN	2	1.2
CKAP5_HUMAN	2	1.3
G3XAN4_HUMAN	2	9
CP51A_HUMAN	2	5.8
C1TM_HUMAN	2	2.6
J3KNJ2_HUMAN	2	17.3
ADCK1_HUMAN	2	6.5
ABD12_HUMAN	2	6.9
FUBP2_HUMAN	2	3.9
CCD47_HUMAN	2	2.1
DDX27_HUMAN	2	2.9
C9JJV6_HUMAN	2	22.4
B7ZLW7_HUMAN	2	2.1
MAGT1_HUMAN	2	5.7
E9PDU5_HUMAN	2	4
F8VZ44_HUMAN	2	6.2
DDX18_HUMAN	2	4.6
ATX10_HUMAN	2	5.4
SEC63_HUMAN	2	3.9
E9PL38_HUMAN	2	10.3
E9PI08_HUMAN	2	7.5
H0YBW4_HUMAN	2	14.8
YTHD2_HUMAN	2	4.3

Supplementary Table 2, FAF1 protein phosphorylation

A4	Sequence	Modifications	Area	PEP	IonScore
High	KsPMMPENAENEG DALLQFTAEFSSR	S2(Phospho)	0.000E0	0.0001333	55
High	TPsGEFLER	S3(Phospho)	7.120E7	0.002514	40
Medium	RSsPAQTR	S3(Phospho)	1.143E7	0.2473	11

Supplementary Table 3, Primers list

	Forward	Reverse
Twist1	5'-GCGCTGCGGAAGATCATC-3'	5'-AGGGTCTGAATCTTGCTCAGCTT-3'
Twist2	5'-CACGCTGCCCTCTGACAAG-3'	5'-CTGCAGGACCTGGTAGAGGAA-3'
Snail1	5'-CCCCAATCGGAAGCCTAACT-3'	5'-GCTGGAAGGTAAACTCTGGATTAGA-3'
Snail2	5'-CAGCTACCCAATGGCCTCTCT-3'	5'-GGACTCACTCGCCCCAAA-3'
Snail3	5'-GCCTGGCCTCACACTTGGT-3'	5'-GGACTTCTTTGGTTCTGATTGGA-3'
Tnfsf10	5'-GCTCTGGGCCGCAAAATA-3'	5'-AGGAATGAATGCCCACTCCTT-3'
Zeb1	5'-TGTGAATGGGCGACCAAGA-3'	5'-GTGGGACTGCCTGGTGATG-3'
Zeb2	5'-AAGATAGGTGGCGCGTGTTT-3'	5'-GACTGACGTGTTACGCCTCTTCTAA-3'
Id1	5'-GGACGAGCAGCAGGTAAACG-3'	5'-TGGGCACCAGCTCCTTGA-3'
RhoA	5'-CGGTCCTCCGTCGGTTCT-3'	5'-GAGACGAAGGCGGGTAGCT-3'
Plaur	5'-CCAATCCTGGAGCTTGAAAATC-3'	5'-TCCCCTTGCAGCTGTAACACT-3'
Pthrp	5'-CCGCCTCAAAAGAGCTGTGT-3'	5'-GGATGGACTTCCCCTTGTCA-3'
Runx1	5'-CCTACCACAGAGCCATCAAAATC-3'	5'-TCATCTAGTTTCTGCCGATGTCTT-3'
Runx2	5'-GCACAAGTAAATCATTGAACTACAGAAA-3'	5'-GAAGCCTGGCGATTTAGAGTTTT-3'
Runx3	5'-CAGGCCCCAGAGAAGATGAG-3'	5'-GCCGGTCTGTAGGTGCTTTC-3'
FN1	5'-TCGCCATCAGTAGAAGGTAGCA-3'	5'-TGTTATACTGAACACCAGGTTGCAA-3'
Acta2	5'-TCCTCCCTTGAGAAGAGTTACGA-3'	5'-ACGTTCATTTCCGATGGTGATC-3'
CDH2	5'-TGGGAATCCGACGAATGG-3'	5'-GCAGATCGGACCGGATACTG-3'
VIM	5'-GGCTCGTCACCTTCGTGAAT-3'	5'-TCTCAATGTCAAGGGCCATCT-3'
MMP2	5'-TGAGCTATGGACCTTGGGAGAA-3'	5'-CCATCGGCGTTCCCATAC-3'
MMP3	5'-AGAGGCATCCACACCCTAGGT-3'	5'-TATCAGAAATGGCTGCATCGAT-3'
MMP9	5'-GGACGATGCCTGCAACGT-3'	5'-CAAATACAGCTGGTTCCCAATCT-3'
MMP14	5'-GGCTCGAGCATTCCAGTGA-3'	5'-GCACAAAATTCTCCGTGTCCAT-3'
Col1a1	5'-CTGTTCTGTTCCTTGTGTAACTGTGTT-3'	5'-TGCCCCGGTGACACATC-3'
Col1a2	5'-CTGAAGTCTCTCAACAACCAGATTG-3'	5'-TGTGCGAGCTGGGTTCTTT-3'
ITGA5	5'-GCACCAACAAGAGAGCCAAAG-3'	5'-CTCCCGCTGCAAGAAAGTCT-3'
MYH9	5'-CTCAGGGCTCATCTACACCTATTCA-3'	5'-TGTACATTTCCACAATCTCTTCAGAGTA-3'
CTGF	5'-AGCCGCCTGTGCATGGT-3'	5'-TTTTGCCCTTCTTAATGTTCTCTTC-3'
CXCR4	5'-CCAGAAGAAACTGAGAAGCATGAC-3'	5'-AGAGGAGGTCGGCCACTGA-3'
CCL22	5'-TGCCCTGGGTGAAGATGATT-3'	5'-AAGGCCACGGTCATCAGAGTA-3'
HES1	5'-ATCCGGAGCTGGTGCTGAT-3'	5'-TCCAGGACCAAGGAGAGAGGTA-3'
IL11	5'-CCTAAAGGGAAATACGCCAAAA-3'	5'-CACCCACAATCCCACCTCTCT-3'
SERPINE1	5'-GAGCTTTTGTGTGCCTGGTAGA-3'	5'-GGCAGGCAGTACAAGAGTGATG-3'
VEGFA	5'-ACGAGGGCCTGGAGTGTGT-3'	5'-TGAGGTTTGATCCGCATAATCTG-3'
ELF5	5'-GGGAATTTGTACGAGACCTGCTT-3'	5'-TCCCTATCTTCCCATTCCAGAA-3'
CDH1	5'-ACAGCCCCGCCTTATGATT-3'	5'-TCGGAACCGCTTCCTTCA-3'
FAF1	5'-TCATCCAGCTTCAGCTCCTACTT-3'	5'-ATCTGCCTGGATGGCATTACA-3'
CXCL11	5'-CACTTCTTTCCCCAAACATCATG-3'	5'-AACAAACCAAATGATGCATAAGAATG-3'
IL6	5'-TGCCTGCGTCCGTAGTTTC-3'	5'-GCTCCCTCTCCCTGTAAGTCTTG-3'
ATF3	5'-CCGCCTTTCATCTGGATTCTAC-3'	5'-AGACACTGCTGCCTGAATCCTAA-3'

JUNB	5’-GCCCGGATGTGCACTAAAAT-3’	5’-CCGTATCCCGTAGCTGTGTATG-3’
TEAD2	5’-CCGAAGGAAATCAAGGGAAAT-3’	5’-GGAAAGCCTTGTCCTTGGAAAC-3’
IGFBP1	5’-TTGGGACGCCATCAGTACCTA-3’	5’-TTTTTTGATGTTGGTGACATGGA-3’
mPAI1	5’-CCGTGGAACAAGAATGAGATCAG-3’	5’-CTCTAGGTCCCGCTGGACAA-3’
mIL11	5’-TGCTGACAAGGCTTCGAGTAGA-3’	5’-CGGCGCAGCCATTGTAC-3’
mCDH2	5’-CACAGCCACAGCCGTCATC-3’	5’-GCAGTAAACTCTGGAGGATTGTCA-3’
mZEB2	5’-GCTGAAGATGATAGCCTTGCAAA-3’	5’-AGCAGCCCTTGGCTCATG-3’
mCTGF	5’-TCCCGAGAAGGGTCAAGCT-3’	5’-TCCTTGGGCTCGTCACACA-3’
mSMAD7	5’-TGGATGGCGTGTGGGTTTA-3’	5’-TGGCGGACTTGATGAAGATG-3’
mSLUG	5’-GCCTGGGTGCCCTGAAG-3’	5’-TTGCAGACACAAGGCAATGTG-3’
mCXCR4	5’-TCGGCAATGGATTGGTGAT-3’	5’-CCGTCATGCTCCTTAGCTTCTT-3’
mZEB1	5’-CCTGACCTGCTGTCGTTCTTT-3’	5’-AGCTCTAGGATGTAATGCCCACTT-3’
mSNAIL	5’-TCCTGCTTGGCTCTCTTGGT-3’	5’-CGGGTACAAAGGCACTCCAT-3’
mIGFBP1	5’-TGGCCCACACTCGGAGAT-3’	5’-CCAGCAAATGTGCATCAGACA-3’
mCDH1	5’-GGCTGGCTGAAAGTGACACA-3’	5’-ACACGGCATGAGAATAGAGGATGTA-3’
m-18S	5'-GTAACCCGTTGAACCCCATT-3'	5'-CCATCCAATCGGTAGTAGCG-3'

Supplementary Reference

1. Rhodes, D.R. *et al.* ONCOMINE: a cancer microarray database and integrated data-mining platform. *NEOPLASIA* 6, 1-6 (2004).
